# The complete chloroplast genome sequence of medicinal plant *Conyza blinii* H.Lév

**DOI:** 10.1080/23802359.2016.1198998

**Published:** 2017-02-03

**Authors:** Tianrun Zheng, Moyang Liu, Tao Wang, Hui Chen

**Affiliations:** College of Life Science, Sichuan Agricultural University, Ya’an, Sichuan, China

**Keywords:** Chloroplast genome, *Conyza blinii*, medicinal plant

## Abstract

*Conyza blinii* is a widely used medicinal plant in the southwest of Sichuan with great economic value. Its chloroplast (cp) genome was 152,503 bp in length with the typical quadripartite structures of the large (LSC, 84,210 bp) and small (SSC, 18,257 bp) single-copy regions, separated by a pair of inverted repeats (IRs, 25,018 bp). The genome contains 114 encoding genes, including 80 protein-coding genes, 30 tRNAs and 4 rRNAs. The overall GC content of the whole cp genome is 37.4% which is similar to the other reported asteridae cp genomes.

Chloroplasts (cps) are indispensable plant cell organelles that conduct photosynthesis in the presence of daylight. The chloroplast genome in land plants is a circular molecule ranging in size from 76 to 217 kb with a highly conserved structure of two copies of large inverted repeats (IR) detached by small (SSC) and large (LSC) single-copy regions. Characteristic of the cp genome have been used comprehensively as a tool to investigate evolutionary relationships and molecular phylogenetic of seed plants (Saski et al. 2005; Lee et al. 2006).

*Conyza blinii* is a biennial herbaceous plant, belongs to *Asteraceae* which is a larger family in angiosperms, and has been used in folk medicine for a long time in the south-west of China. With the further study, a new neoclerodane diterpenelactone, blinin, was isolated from the whole plant of *C. blinii*. It is also reported to have a decisive role in pharmaceutical research (Yang et al. 1989).

This study reports the sequencing of the *C. blinii*, and the plants in the wild were collected in Panzhihua (Coordinate: 26.562222N, 101.796389E; Altitude: 1910 m). The whole cp genome was sequenced using HiSeq 2000, Illumina and assembled into the complete cp genome using Sequencher version 4.10. DualOrganellarGenoMe Annotator (Austin, TX) (Wyman et al. 2004) was utilized to annotate the cp genome with BLASTX and BLASTN identifying the location of encoding genes and RNAs. The complete genome sequence was submitted to GenBank under the accession number KX085421.

The total size of complete cp genome was 152,503 bp with 37.4% GC content, consisted of a typical quadripartite structures of one LSC (84,210 bp), one SSC (18,257 bp), and two IRs (25,018 bp). The GC content of the SSC (43%) was higher than that of LSC and IR (35.3% and 31.4%, correspondingly), which has significant differences compared to the majority of angiosperm (Liu et al. 2013; Zhang et al. 2014; Choi & Park 2015; Curci et al. 2015; Lu et al. 2015). Usually, the IR region has the highest GC content, mainly caused by the the high GC content of the four ribosomal RNA (rRNA) genes. A total of 114 genes were successfully annotated, including 80 protein-coding genes, 30 tRNA genes and 4 rRNA genes; and 21 out of 114 genes were duplicated in inverted repeat regions. There were 17 intron-containing genes, almost all of which were single-intron genes except for *ycf*3 and *clp*P with two introns separately. Exceptionally, *rps*12 was a reverse splicing gene, of which the 5′ end exon located in LSC region and 3′ end exon was in IR region. The phylogenetic relationship of *C. blinii* was deduced by comparing it with the other 18 chloroplast genomes downloaded from Genbank (Figure 1).

**Figure 1.**
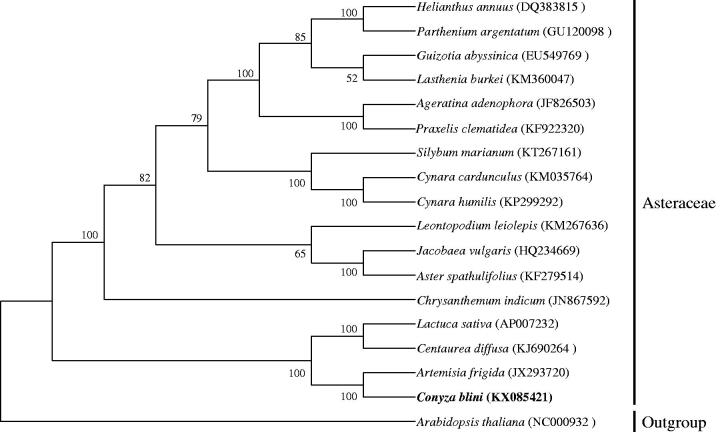
ML tree based on the *C. blinii* chloroplast genomic sequence compared with 18 sequences obtained from NCBI.
